# Advanced biomanufacturing and evaluation of adeno-associated virus

**DOI:** 10.1186/s13036-024-00409-4

**Published:** 2024-02-15

**Authors:** Kai Chen, Seulhee Kim, Siying Yang, Tanvi Varadkar, Zhuoxin Zora Zhou, Jiashuai Zhang, Lufang Zhou, Xiaoguang Margaret Liu

**Affiliations:** 1 Department of Chemical and Biomolecular Engineering, The Ohio State University (OSU), 151 W Woodruff Ave, Columbus, OH 43210 USA; 2 Department of Biomedical Engineering, The Ohio State University, 140 W 19th Ave, Columbus, OH 43210 USA; 3 Comprehensive Cancer Center (CCC), The Ohio State University, 650 Ackerman Rd, Columbus, OH 43202 USA

**Keywords:** Adeno-associated virus (AAV), Biomanufacturing, Bioproduction, Purification

## Abstract

Recombinant adeno-associated virus (rAAV) has been developed as a safe and effective gene delivery vehicle to treat rare genetic diseases. This study aimed to establish a novel biomanufacturing process to achieve high production and purification of various AAV serotypes (AAV2, 5, DJ, DJ8). First, a robust suspensive production process was developed and optimized using Gibco Viral Production Cell 2.0 in 30–60 mL shaker flask cultures by evaluating host cells, cell density at the time of transfection and plasmid amount, adapted to 60–100 mL spinner flask production, and scaled up to 1.2–2.0-L stirred-tank bioreactor production at 37 °C, pH 7.0, 210 rpm and DO 40%. The optimal process generated AAV titer of 7.52–8.14 × 10^10^ vg/mL. Second, a new AAV purification using liquid chromatography was developed and optimized to reach recovery rate of 85–95% of all four serotypes. Post-purification desalting and concentration procedures were also investigated. Then the generated AAVs were evaluated in vitro using Western blotting, transmission electron microscope, confocal microscope and bioluminescence detection. Finally, the in vivo infection and functional gene expression of AAV were confirmed in tumor xenografted mouse model. In conclusion, this study reported a robust, scalable, and universal biomanufacturing platform of AAV production, clarification and purification.

## Introduction

Since adeno-associated virus (AAV) was first identified in 1965, hundreds of variants have been isolated from adenovirus stocks or primate tissues [1]. Different serotypes of AAV1-10 could preferentially transduce or induce specific types of cells or tissues, enabling organ-based gene delivery [2–4]. Various recombinant AAVs (rAAVs) have been constructed to transduce a wide range of living cells (dividing and non-dividing) and deliver genes of interests [5, 6]. rAAV constitutes 20–25-nm non-envelop protein capsid (virion protein 1 [VP1], VP2 and VP3) and < 4.9-kb genome of single-stranded DNA (ssDNA), including the gene of interest, flanked by inverted terminal repeats [7, 8]. Novel engineered chimeric AAV capsids, such as AAV-DJ, -DJ8 and others, have been constructed by Kay [9], Samulski [10] and Schaffer [11] Laboratories to escape AAV neutralization by pre-existing serum antibodies, increase in vivo infection efficiency, and enhance circulation stability.

Several AAV-mediated gene therapies [12, 13], including Glybera (AAV1 delivering S447X, withdrawn from market) for lipoprotein lipase deficiency treatment, Roctavian (AAV5 carrying clotting factor VIII) for adults with severe hemophilia A, Hemgenix (AAV5 delivering clotting factor IX) for hemophilia B, Luxturna (AAV2 carrying functional RPE65 gene) to treat inherited retinal disease, Zolgensma (AAV9 carrying SMN1) for children below two years old with spinal muscular atrophy, and Elevidys (AAV delivering micro-dystrophin protein) for ambulatory pediatric patients, have been approved so far. As compared to conventional nanoparticle-mediated gene delivery vehicles, rAAV has advantages of high infection rate, long-term transgene expression, low immunogenicity, and minimal toxicity in clinical applications [1, 14, 15]. Over 130 AAV-delivered gene therapies have been evaluated in clinical trials during last two decades [16] and hundreds of clinical trials are on-going to treat Alzheimer, Parkinson, and other diseases [17–20]. Due to the promising clinical and pre-clinical achievements of these gene therapies, an advanced AAV biomanufacturing procedure with high productivity, quality and recovery rate for multiple serotypes is highly needed.

Literature has reported several suspensive bioproduction processes of AAV expression vectors using HEK cells and triple-plasmid transfection [21]. For instance, Daniel et al. have reported a polyethylenimine (PEI)-mediated transfection of suspensive HEK 293 cells to produce AAV2/8 and AAV2/9 carrying green fluorescent protein (GFP) with titer of 2 × 10^8^ vg/mL at 10–30 mL scale [22]. The purification procedure using iodixanol gradient ultracentrifugation and immunoaffinity chromatography with POROS CaptureSelect resin has generated recovery rate of 35.6% and 17.9%, respectively. Grieger et al. have well adapted HEK 293 cell in suspension culture, transfected with three plasmids and PEI Max, and produced AAV serotypes 1–6, 8 and 9 in 30-mL shaker flask culture and 2 or 4-L WAVE bioreactor culture with titer of 0.9–3.5 × 10^10^ vg/mL [23]. The ion exchange purification using 5-mL HiTrap Q HP column generated rAAV with purification recovery of 39–49%. In another study, the suspensive HEK 293 T cells have been transfected with PEI Max and three plasmids to generate AAV serotypes of 1, 2, 5, 8 and 9 in 30-mL or 1-L shaker flask cultures, which achieved final titer of > 1 × 10^11^ vg/mL [24]. The affinity purification using AVB-Sepharose with POROS CaptureSelect has yielded an estimated recovery of 27.9–76.9%. In our previously developed AAV biomanufacturing process, suspensive HEK 293F cells and PEI transfection reagent or liposomes produced up to 7.86 × 10^9^ vg/mL of chimeric AAV-DJ8 in 30–450 mL of shaker flask or spinner flask cultures [25]. To meet the increasing demand of clinical materials, a more advanced biomanufacturing platform with high AAV productivity and recovery rate is needed.

This study aimed to develop a robust and scalable biomanufacturing platform to produce AAVs in stirred-tank bioreactor, purify various AAV serotypes using liquid chromatography, and improve overall recovery rate in the procedures of clarification, purification and post-purification operations. The effects of host cell, key transfection parameters (e.g., viable cell density, transfection reagent, ratio of plasmid DNA: cell), production scale, clarification strategy, purification column with different loading and elution conditions, and desalting and concentration method were evaluated and compared. Importantly, this study evaluated the scale-up robustness of our developed bioproduction and purification processes, which is essential to adapt it from Good Laboratory Practice (GLP) to future Good Manufacturing Practice (GMP) production. The generated AAVs were fully evaluated in terms of capsid protein expression, morphology, transduction capability, tissue-specific infection, and functional expression of delivered gene. The advanced AAV biomanufacturing reported in this study could benefit the future GMP production of multiple AAV serotypes and their further pre-clinical and clinical evaluations.

## Results and discussion

### Advanced biomanufacturing of AAV

**Fig. 1:**
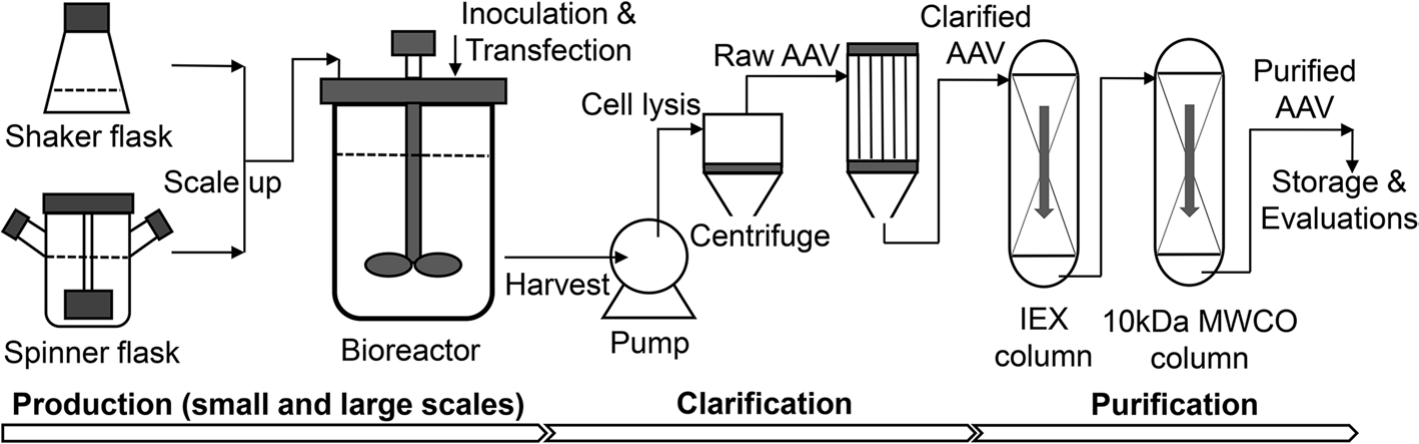
Process flow diagram (PFD) of the advanced AAV biomanufacturing platform, including production, clarification and purification

The process flow diagram (PFD) of an advanced AAV biomanufacturing was developed in this study, including suspensive production, bioproduction scale up, clarification, liquid chromatography purification and scale up, post-purification process, storage and evaluations (Fig. 1). The AAV production process development was performed in shaker flasks at scale of 30–100 mL and in spinner flasks with 60–100 mL of cultures. The production process in 1.2–2.0 L of stirred-tank bioreactors with process parameter control could be applied to pilot plant production and possible large-scale manufacturing production. As detailed later, the key production parameters identified in this study include host cell selection, transfection condition, and agitation speed. Two-step universal separation process using anionic exchange chromatography and ultrafiltration has been developed to purify multiple AAV serotypes. The post-purification desalting and concentration procedures have also been investigated. This study reported an advanced generic AAV biomanufacturing process of production, clarification and purification. Importantly, the developed platform is robust, scalable, and applicable to cover multiple (if not all) serotypes.

### Development of suspensive AAV production and clarification

**Fig. 2:**
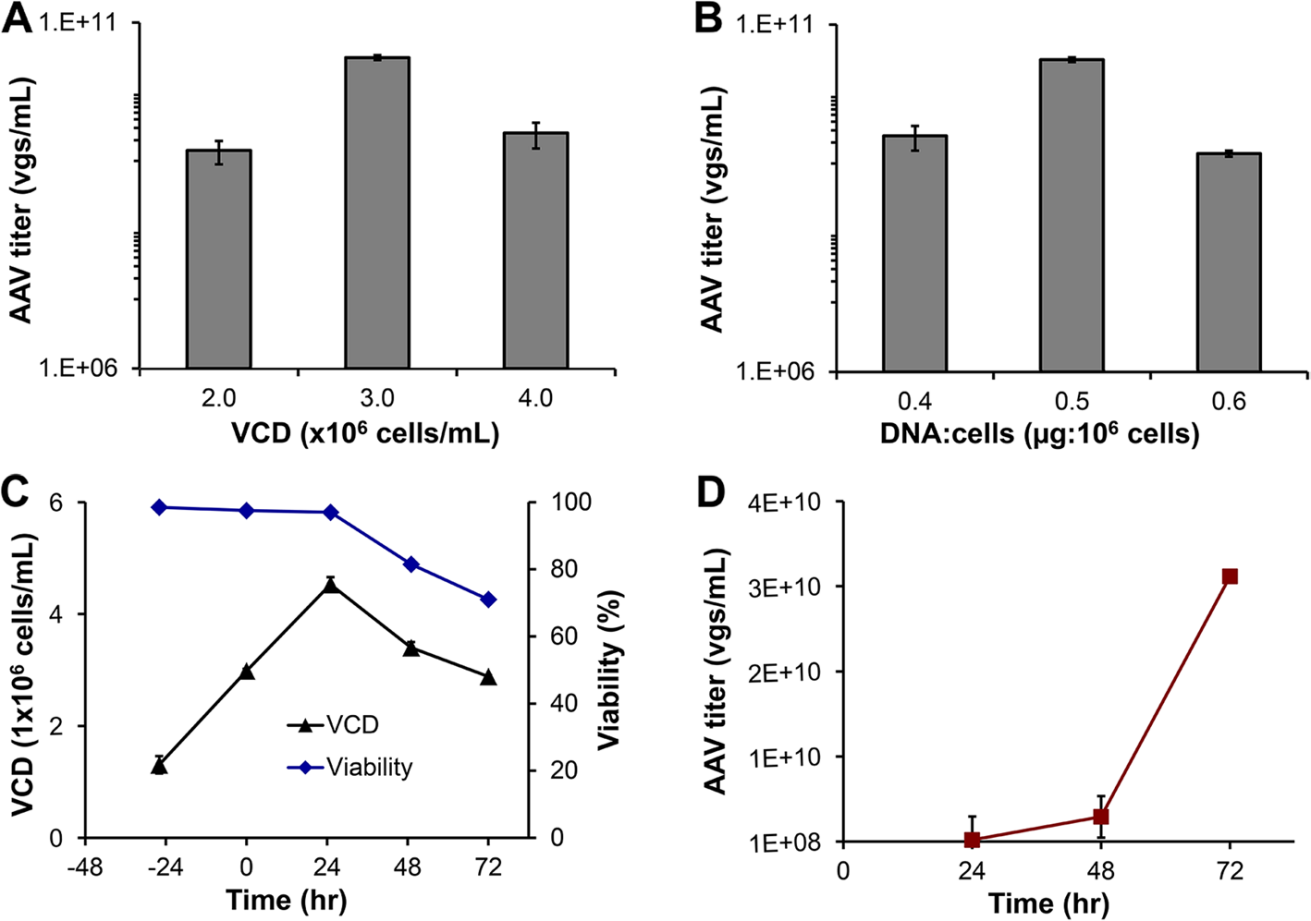
Development of 3-day suspensive AAV production in small-scale shaker flask. **A** Evaluation of transfection VCD of 2.0, 3.0, and 4.0 × 10^6^ cells/mL. **B** Evaluation of ratio of plasmid DNA and VPC cells including 0.4, 0.5 and 0.6 μg/1 × 10^6^ cells. **C** Viable cell density and viability of VPC pre- and post-transfection with maximal VCD of 4.53 × 10^6^ cells/mL and harvest viability of 71% with optimal transfection conditions. **D** Volumetric productivity of AAV with final titer of 3.13 × 10.^10^ vg/mL with optimal transfection conditions. VPC cells were cultivated in 30-mL viral production medium supplemented with 6 g/L of glucose and 4 mM of GlutaMax at 37 °C, 8% CO_2_, and 130 rpm. The production process could be applied to four serotypes (AAV2, 5, DJ, and DJ/8)

We first compared two suspensive host cells, i.e. HEK 293F and VPC, in shaker flask production at 37 °C, 8% CO_2_ and 125 rpm. Both seed and production cultures showed that VPC cells had significantly lower cell clumping than HEK 293F. The VCD of VPC reached > 4.0 × 10^6^ cells/mL on Day 1 post transfection, followed with VCD and viability dropping to ~ 3.4 × 10^6^ cells/mL and 82% and AAV-DJ8 titer increased significantly from Day 2 (Fig. 2). AAV was harvested at 72 h post transfection with VCD of 2.8 × 10^6^ cells/mL and cell viability of 70–80% in shaker flask. The dynamic production profile revealed a significant increase of AAV titer from Day 2 to Day 3. Similar cell growth and AAV productivity were observed in the productions of AAV2, AAV5 and AAV-DJ (cell culture profiles not shown). As summarized in Table 1, the volumetric productivity of AAV-DJ8 using the same triple plasmids, pAAV-NLuc-GOI (~ 3.9 kb), pAAV Rep-Cap and pHelper, was 0.50–0.53 ± 0.08 × 10^10^ vg/mL by HEK 293F and 2.40 ± 0.06 × 10^10^ vg/mL by VPC cells under respective optimal transfection conditions. It is obvious that VPC produced about 5-fold higher AAV in shaker flask than HEK 293F. Therefore, the process development and scale up in this study used VPC.

**Table 1:** Summary of AAV biomanufacturing development

**Host cells**	**Production container**	**Scale** (mL)	**AAV serotype**	**Transfect. reagent**	**Transfection VCD** (c/mL)	**DNA:cell (µg:10** ^**6**^ **)**	**Productivity (10** ^**10**^ **vg/mL)**	**Purification**	**Purify recovery**	**Desalt/concentrate**	**Overall recovery**
HEK 293F	Shaker flask	30–60	AAV-DJ8	Liposomes	0.4 × 10^6^	1.6:1	0.53 ± 0.08	10 kDa ultrafilter (UF)			N/A
	Spinner flask	60–100	AAV-DJ8	Liposomes	0.4 × 10^6^	1.6:1	0.50 ± 0.08	10 kDa UF			N/A
VPC	Shaker flask	30–60	AAV2	AAV-MAX	3 × 10^6^	0.5:1	7.88 ± 0.39	Anion IEX (HPQ)	40–60%	G-25/Vacuum	30–50%
								HPQ	40–60%	PES UF	> 50%
								Affinity (AVB), Sepharose (IEX)	< 5%	N/A	< 5%
		30–60	AAV5	AAV-MAX	3 × 10^6^	0.5:1	2.97 ± 0.13	HPQ	40–60%	G-25/Vacuum	30–50%
		30–60	AAV-DJ8	AAV-MAX	2 × 10^6^	0.5:1	0.14 ± 0.05	HPQ	40–60%	G-25/Vacuum	30–50%
					4 × 10^6^	0.5:1	0.25 ± 0.13				
					3 × 10^6^	0.4:1	0.25 ± 0.09				
					3 × 10^6^	0.6:1	0.14 ± 0.01				
		30–60	AAV-DJ8	AAV-MAX	3 × 10^6^	0.5:1	2.40 ± 0.06	Anion IEX (aPrime 4A)	85–95%	PES UF	> 80%
								HPQ	40–60%	G-25/Vacuum	27–34%
								HPQ	40–60%	PES UF	52–64%
								AVB, IEX	< 5%	N/A	< 5%
		30–60	AAV-DJ	AAV-MAX	3 × 10^6^	0.5:1	5.60 ± 5.14	HPQ	> 90%	Cellulose UF	65–91%
	Spinner flask	60–100	AAV-DJ8	AAV-MAX	3 × 10^6^	0.5:1	0.41 ± 0.20	HPQ	> 30%	G-25/Vacuum	10–20%
	Stirred-tank bioreactor	1,200	AAV-DJ	AAV-MAX	3 × 10^6^	0.5:1	8.14 ± 1.91	HPQ	40–60%	Dialysis/Vacuum	18–33%
		1,500–2,000	AAV-DJ8	AAV-MAX	3 × 10^6^	0.5:1	7.52 ± 0.49	aPrime 4A	> 85%	G-25/Vacuum	> 80%

All data are presented as mean ± standard error of the mean (SEM). *n* = 2–3

Our previous study showed that the cell density at the time of transfection and amount of plasmid DNA are other two key transfection parameters to improve AAV production [25]. Therefore, we evaluated the effects of cell density at the time of transfection (2.0, 3.0, and 4.0 × 10^6^ cells/mL) and ratio of total plasmid DNA/VPC cells (0.4, 0.5, and 0.6 μg/million cells) in shaker flask productions. As presented in Fig. 2A and B, the optimal transfection VCD is 3.0 × 10^6^ cells/mL and plasmid DNA: VPC ratio is 0.5 µg: 10^6^ cells, which generated final AAV titer of 5.6–10.0 × 10^10^ vg/mL. Therefore, our scaling up evaluation and purification development studies used the optimal transfection VCD and plasmid DNA amount identified here.

Then the optimal suspensive production process was validated with four AAV serotypes, using pAAV2, 5, DJ and DJ8 Rep-Cap, expression vector with ~ 3.9 kb of inserted genes and pHelper, in shaker flask and/or bioreactor cultures at the developed conditions. The optimal transfection formulation, i.e. pAAV expression: pAAV Rep-Cap: pHelper ratio of 1:1:3, DNA: cell ratio of 0.5 µg:1 million cells, 10% viral-plex buffer and 0.6% AAV-MAX transfection reagent, and supplement of 0.3% booster and 1% enhancer, was applied. The qPCR titration of intracellular AAV showed similar range of productivity of 7.88 ± 0.39, 2.97 ± 0.13, 2.40 ± 0.06, and 5.60 ± 5.14 × 10^10^ vg/mL for AAV2, AAV5, AAV-DJ8, and AAV-DJ, respectively (Table 1). These results demonstrated that the suspensive AAV production process can be used to generate multiple serotypes.

Furthermore, we investigated and compared several raw AAV clarification strategies, including direct lysis of cell culture broth and lysis of cell pellets after centrifugation. The direct lysis by adding AAV-MAX lysis buffer and other supplements (MgCl_2_ and benzonase) and incubating the lysis mixture at 37 °C was time-consuming (2–6 h), and also had poor cell lysis efficiency in some batches which could be caused by culture variations. Then we tested the strategy of centrifugation to collect cell pellets followed with two lysis options as detailed in Section “AAV clarification”. Our results demonstrated that both strategies, i.e. incubation at 37 °C and repeated freeze–thaw cycles, achieved 95–100% VPC lysis. The lysis of culture broth enables direct collection of raw AAV from most productions tested in this study, but cell pellet lysis could achieve high AAV release efficiency (as confirmed with cell lysis rate), reduce lysis reagent amount and simplify clarification operation in bioreactor-based production.

### Bioproduction scale-up

**Fig. 3:**
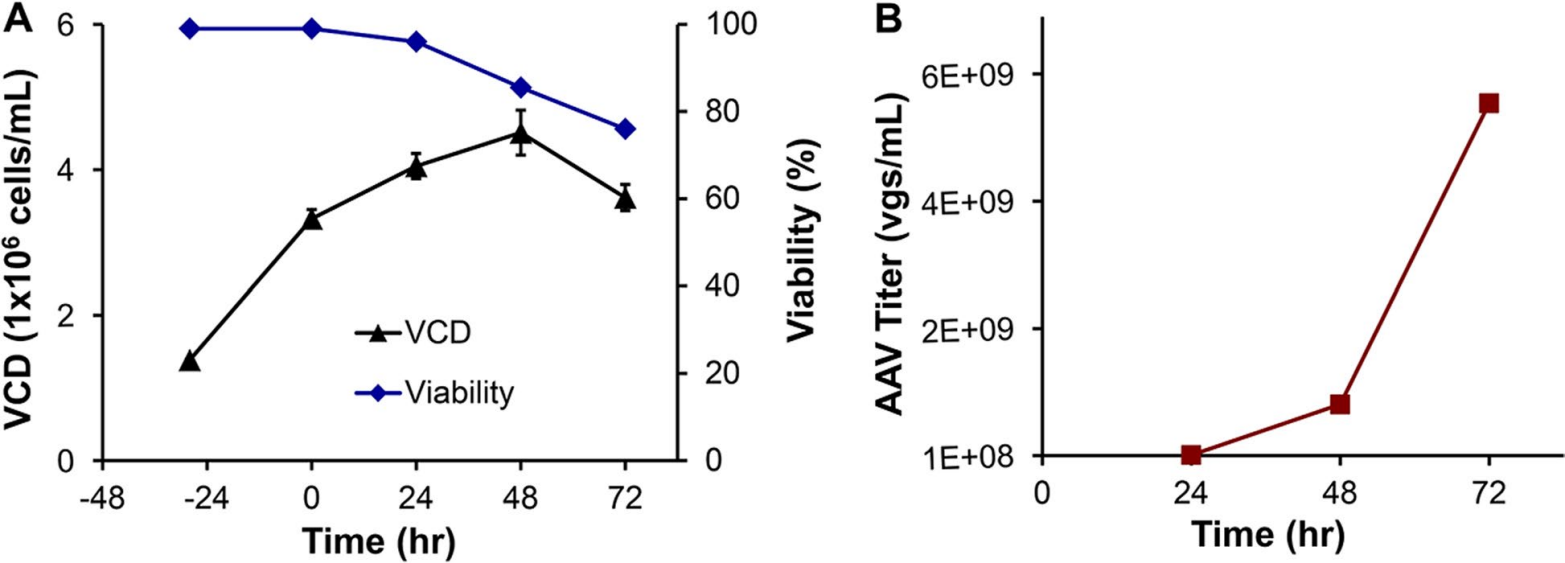
AAV production in spinner flask. **A** Kinetic profile of VPC cell growth with peak VCD of 4.51 × 10^6^ cells/mL and harvest viability of 76%. **B** AAV production with final titer of 0.59 × 10^10^ vg/mL. Spinner flaks cultures were carried out at 37 °C, 8% CO_2_, and 230 rpm using AAV-DJ8 as model virus

Before scaling up shaker flask production process to stirred-tank bioreactor, AAV production was evaluated in 250-mL spinner flask with working volume of 60–100 mL. The agitation speeds of 75, 100, 125 and 150 rpm were tested. The low agitation speed caused significant cell aggregation and shortened culture longevity. The AAV productions in spinner flask presented in Table 1 were performed at 37 °C, 210 rpm and 8% CO_2_. As compared to shaker flask, spinner flask production reached maximal VCD of 4.3–4.6 × 10^6^ cells/mL on Day 2 and VPC cells containing AAV were harvested at viability of 70–80% (Fig. 3). Similar to shaker flask cultures, AAV titer was significantly increased from Day 2 to Day 3 in spinner flask. It was observed that spinner flask production was less than 20% of that in shaker flask, i.e., 0.41 vs 2.40 × 10^10^ vg/mL. These results suggested that the suspensive transfection and AAV production in stirred tank is feasible, but the process parameters need further optimization for high productivity.

**Fig. 4:**
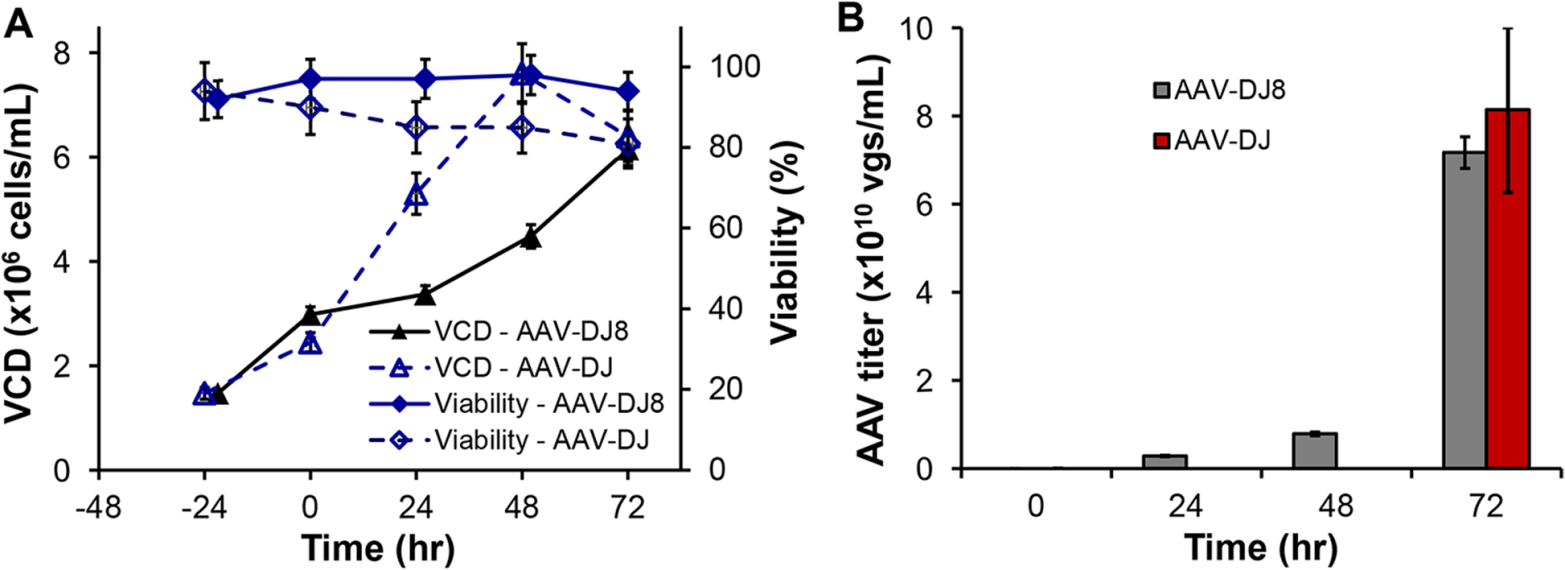
Scaled up AAV production in stirred-tank bioreactor. **A** VPC cell growth profile with peak VCD of 6.15 × 10^6^ cells/mL (AAV-DJ8) or 7.60 × 10^6^ cells/mL (AAV-DJ). VPC had better and healthier cell growth in bioreactor. **B** AAV concentration reached 7.17 × 10^10^ vg/mL (AAV-DJ8) or 8.14 × 10^10^ vg/mL (AAV-DJ). The 1.2–2.0 L of production cultures were performed in 2.5-L bioreactor with automatically controlled process parameters of 37 °C, pH 7.0, 210 rpm and DO 40%

Next, we investigated the process scale up to stirred-tank bioreactor using seed cultures from shaker flask or spinner flask. Both strategies showed similar cell growth and AAV production, so all bioreactor productions presented in this study used shaker flask seed cultures. As shown in Fig. 4, the maximal VCD reached 6.15 × 10^6^ cells/mL (AAV-DJ8) or 7.60 × 10^6^ cells/mL (AAV-DJ) and harvest viability was about 90–95% (AAV-DJ8) or 80–85% (AAV-DJ) at 72 h post triple-plasmid transfection, which had different cell growth kinetic profile from those in shaker flask and spinner flask. The production titers of 8.14 ± 1.91 × 10^10^ vg/mL for AAV-DJ and 7.52 ± 0.49 × 10^10^ vg/mL for AAV-DJ8 were obtained on Day 3 in 1.2–2-L bioreactor production at 37 °C, pH 7.0, 210 rpm and DO 40%. It is clear that VPC cell growth was enhanced by ~ 50% and AAV titer was improved by > 100% in stirred-tank bioreactor as compared to shaker flask (Table 1). These process-scaling up data demonstrated that our AAV production process was robust and scalable in bioreactors, which is important to future industrial productions to support clinical trials or potential clinical applications.

### Purification development and scale-up

Multiple commercial columns for AAV purification have been evaluated in this study (Table 1), including Cytiva HiTrap Q Sepharose XL strong anion exchange column, Cytiva Sepharose Fast Flow anion exchange column, Cytiva HiTrap AVB Sepharose column, Bio-Rad Foresight Nuvia HPQ column, and Bio-Rad EconoFit Nuria aPrime 4A. The primary purification method using NGC liquid chromatography equipped with these columns were developed.

**Fig. 5:**
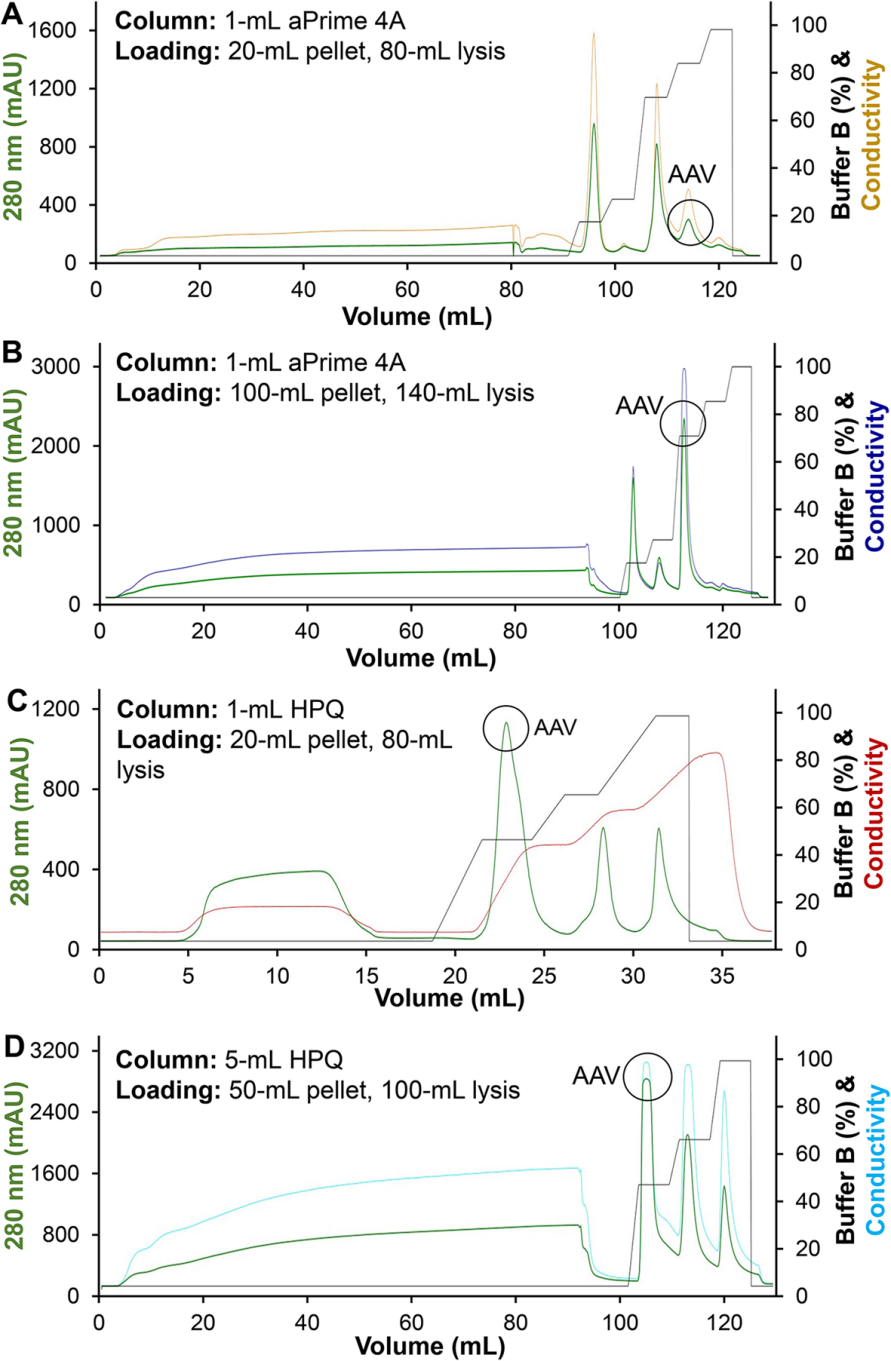
Development and optimization of anion exchange purification using liquid chromatography (LC). The 80–140 mL of cell lysis from 20–100 mL of VPC pellet was loaded to the 1-mL or 5-mL columns. The representative LC profile of AAV-DJ8 was described here but four serotypes of AAV2, 5, DJ and DJ8 were tested using the developed purification strategy. Equilibration buffer: 25 mM Tris–HCl, pH 9.0. Elution Buffer A: 25 mM Tris–HCl, 20 mM NaCl, pH 9.0. Elution buffer B: 25 mM Tris–HCl, 1 M NaCl, pH 9.0. Flow rate: 1.0 mL/min. **A** Stepwise elution of AAV-DJ8, 80-mL AAV lysis from 20-mL VPC pellet, in 1-mL EconoFit Nuvia aPrime 4A column. **B** Stepwise elution of AAV-DJ8, 140-mL AAV lysis from 100-mL VPC pellet, in 1-mL EconoFit Nuvia aPrime 4A column. **C** Stepwise elution of AAV-DJ8, 100-mL AAV lysis from 50-mL VPC pellet, using 1-mL Foresight Nuvia HPQ column, which can be scaled up from 1-mL column to 5-mL and 25-mL columns. **D** Stepwise elution of AAV-DJ8, 100-mL AAV lysis from 50-mL VPC pellet, using 5-mL Foresight Nuvia HPQ column

As shown in Fig. 5, aPrime 4A column achieved purification recovery of > 85% using equilibration buffer A (25 mM Tris–HCl, 20 mM NaCl, pH 9.0) and elution buffers of A and B (25 mM Tris–HCl, 1 M NaCl, pH 9.0). Linear elution (0 ➔ 100% increase of buffer B) in aPrime 4A column did not well separate AAV from other peaks (data not shown). The stepwise elution (0, 15, 25, 70, 85 and 100% of buffer B) at flow rate of 1.0 mL/min well separated AAV peak from other impurities, with high binding rate of 85–95% and elution rate of ~ 100%, using 1-mL aPrime 4A column and pellet lysate from 20-mL culture (Fig. 5A). The binding rate was calculated by titrating raw AAV samples and flow through collection. We further increased the loading amount of raw AAV by using pellet lysate from 100-mL culture in 1-mL aPrime 4A column, which showed that the AAV binding rate was reduced to < 80% although the binding amount was significantly increased (Fig. 5B). The representative chromatography profile of AAV-DJ8 was described in Fig. 5, but four serotypes of AAV2, 5, DJ and DJ8 were tested using the same column, loading and elution conditions, which did not show obvious difference in binding and elution. These results confirmed the robustness and scalability of our primary AAV purification using IEX. Small amount of AAV was detected in flow through and other elution peaks from aPrime 4A column. Further optimization of sample loading and elution conditions (e.g. flow rate and stepwise strategy of buffer B) might be able to increase the overall purification recovery rate.

The stepwise elution (0, 50, 65 and 100% of buffer B) of raw AAV lysis from 20-mL pellet using 1-mL HPQ column showed lower binding and overall recovery rate of 40–60% (Fig. 5C) than aPrime 4A. Furthermore, we scaled up the purification process to a 5-mL pre-packed commercial HPQ column, loaded with AAV lysis from 50-mL pellet (Fig. 5D), and to an in house packed 25-mL column using the same Nuvia HPQ media. The similar binding rate, elution profile and recovery rate were observed in both 5-mL and 25-mL HPQ columns while significant (~ 50%) amount of AAV was detected in flow through and other elution peaks using HPQ column.

The evaluations of other commercial columns showed that the Q Sepharose IEX column had low AAV binding rate (< 5%) and AVB Sepharose affinity column showed weak binding rate (< 5%) of AAV2 and DJ8 using the manufacturer provided purification parameters as detailed in Section “AAV purification”. Taken together, the IEX purification using aPrime 4A column with stepwise elution was identified as the optimal primary purification in this study although further development and optimization is needed in future.

The secondary purification using ultrafiltration and other strategy such as G25 column or dialysis was tested to concentrate and desalt (i.e. buffer exchange) the purified AAV. The AAV2, 5 and DJ8, which were filtered, concentrated and washed with PBS using 100 kDa MWCO PES column following manufacture procedure, showed high recovery rate (> 90%). However, the AAV-DJ elute from IEX column blocked PES column, and 100 kDa MWCO regenerated cellulose column was identified as a suitable column to ultrafiltrate AAV-DJ with high recovery rate of 90%. The alternative strategies are to combine desalting operation using HiTrap G25 column equipped in liquid chromatography system following the manufacture protocol or 20 kDa dialysis cassette with additional ultrafiltration concentration or refrigerated vacuum concentrator. The purified AAVs were aliquoted in formulation buffer of 1 × PBS, 5% Sorbitol and 350 mmol/L NaCl, and stored at -80 °C for long term.

### Quality evaluations of produced AAV

**Fig. 6:**
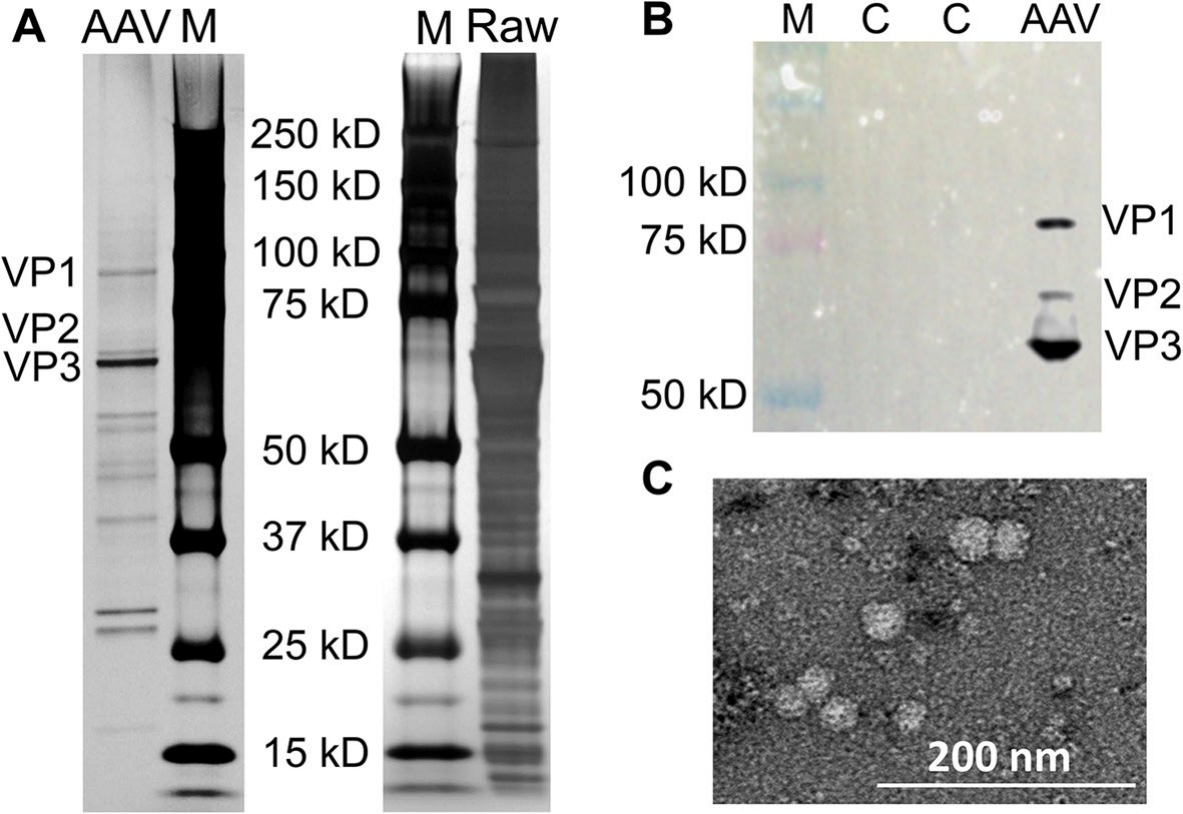
Characterizations of produced AAV.** A** SDS-PAGE of AAV pre-purification and post anion exchange purification. M: marker, and C: negative control protein. **B** Western blot confirmed three AAV capsid proteins: VP1 (87 kDa), VP2 (73 kDa) and VP3 (62 kDa). **C** TEM image of purified AAV. Scale bar: 200 nm

**Fig. 7:**
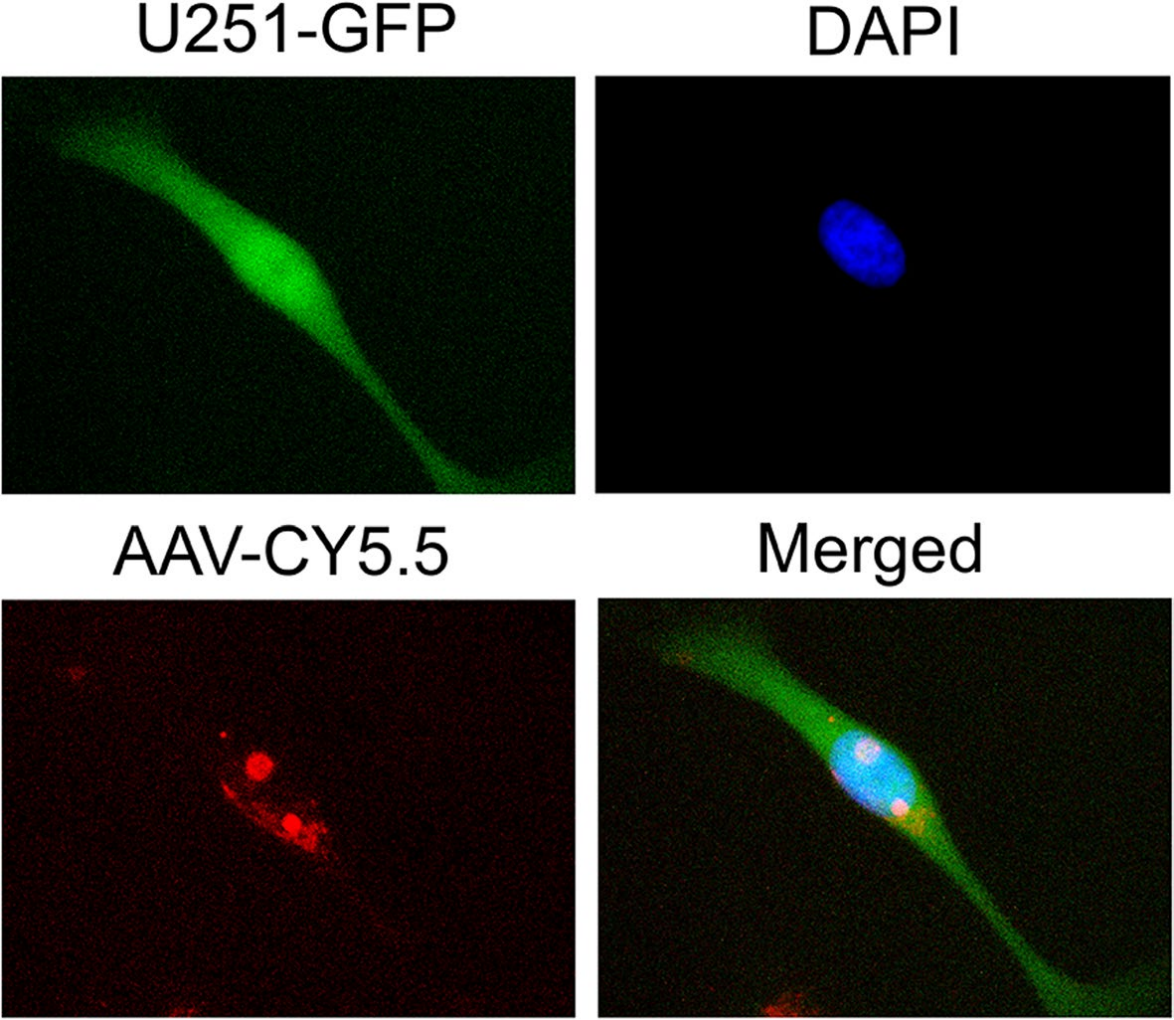
Confocal microscope demonstrating high transduction of AAV, revealed by co-localization of green GFP (U251 cells), blue DAPI (nucleus), and red Sulfo-cyanine 5.5 (AAV). MOI = 5,000

Although the developed biomanufacturing process was validated using four serotypes of AAV, the AAV-DJ8 was applied in the following characterizations or evaluations. To characterize the AAV-DJ8 produced from our developed bioprocess, SDS-PAGE was performed with silver staining and detected three capsid proteins, 87-kDa VP1, 73-kDa VP2 and 62-kDa VP3 (Fig. 6A). Western blotting was carried out to analyze the purified AAV, which confirmed the integrity and expression of all three capsid proteins (Fig. 6B). Moreover, TEM image confirmed the right size and morphology of AAV (Fig. 6C). In addition to high productivity and recovery, transduction capability of functional AAV was also evaluated using live-cell imaging. As described in Fig. 7, glioblastoma U251 cells (green color, GFP labelled) were transduced with Cy5.5-labelled AAV-DJ8 (red color), and confocal microscope imaging demonstrated that AAV accumulated around the DAPI-stained nucleaus (blue color) within 24 h post incubation. These images revealed that our AAV could effectively transduce cells in vitro.

**Fig. 8:**
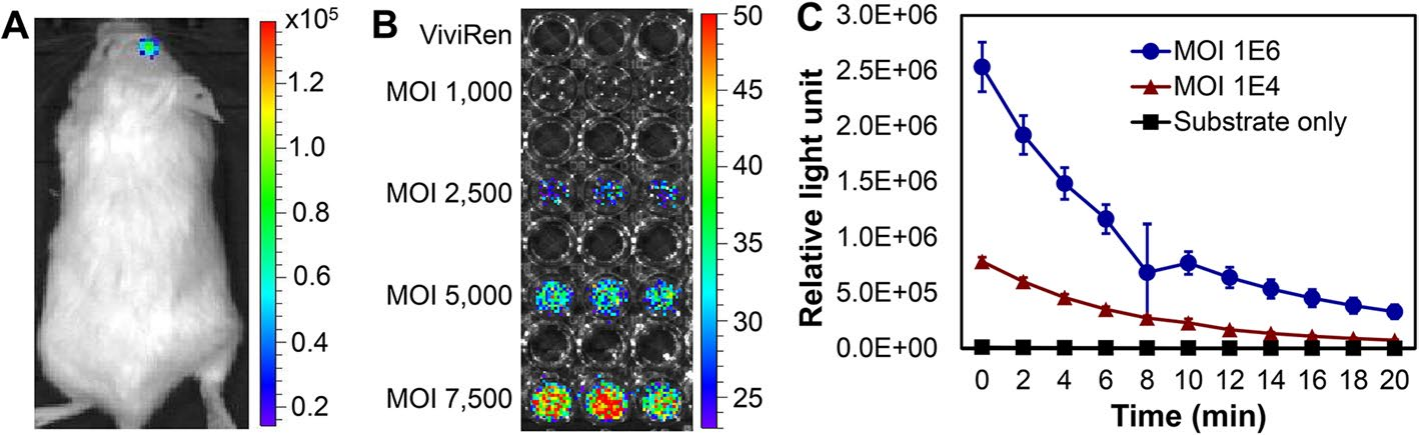
Evaluations of functional gene expression. **A** Live-animal IVIS imaging showed high in vivo expression of AAV-delivered gene. About 0.5 × 10^6^ U251 cells were intracranially injected to NSG mice using stereotactic instrument to develop glioblastoma xenografted models. AAV (1 × 10^11^ vg) and ViviRen (3.7 μg) were injected. **B** In vitro AAV gene expression is dosage (multiplicity of infection, MOI)-dependent. **C** AAV gene expression correlates to MOI, as measured by i3x plate reader

The in vivo AAV induction and functional expression of AAV-delivered gene were tested by intracranially injecting 1 × 10^10^ vg of AAV-DJ8 into the glioblastoma U251 xenograft NSG mouse models. As described in Fig. 8A, the *NLuc* gene was delivered to glioblastoma tumor and functionally expressed to generate bioluminescence in vivo with induction of ViviRen (37 µg, intravenous injection), as detected by live-animal IVIS imaging. This result also confirmed the gene expression in tumor only facilitated with the tumor-specific promoter in AAV expression vector [25]. It was observed that the in vivo NLuc bioluminecence lasted 1–2 h post injection of substrate ViviRen.

Furthermore, we transduced 5 × 10^4^ cells of U251 that were seeded in 96-well plates with AAV-DJ8 at MOIs of 0, 1,000, 2,500, 5,000, and 7,500. Neither MOI of 0 (25 µM of ViviRen only) nor 1,000 generated bioluminescence signal while MOIs of 5,000 and 7,500 had strong bioluminescence (Fig. 8B). Higher MOI of AAV generated stronger bioluminescence than lower MOI in 6-well plate cultures. The dynamic SpectraMax iD3 profiles showed that the bioluminescence signal decreased to minimal levels within 25 min post induction in vitro (Fig. 8C). All these characterization and evaluation data demonstrated that our new biomanfuacturing process generated high-quality AAVs.

### Advantages of our AAV biomanufacturing process

This study developed a novel AAV biomanufacturing procedure with multiple advantages as compared to previously reported production processes. First, high productivity can be achieved in the stirred-tank bioreactor-based production. Second, the developed process is robust and scalable to large-scale biomanufacturing for future pre-clinical and clinical trials. Third, good-purity AAV was generated using the identified ion-exchange columns and developed purification protocols. Fourth, the good-quality AAV produced from the developed process can be used in vitro and in vivo without detected side effects such as fever or immune toxicity. Most importantly, the developed universal biomanufacturing process can be applied to produce and purify different serotypes of AAV (AAV2, 5, DJ and DJ8 in this study).

### Prospective AAV biomanufacturing

This study developed a scalable suspensive AAV production process by evaluating host cell and transfection parameters. The Viral Production Cell (VPC) 2.0 engineered from parental HEK 293F cells by Gibco, which has larger cell size, faster cell growth, and minimal cell clumping at optimal shaking or agitation condition, was applied and enhanced AAV titer by 5 folds as compared to HEK 293F [25]. Compared to adherent HEK 293AAV or 293A, the VPC-based AAV production process is robust and easy to scale up in bioreactor. Moreover, this host cell showed high resistance to shear force and could directly inoculate the production medium in bioreactor using the seed cultures from shaker flask without any adaptation.

One of the key parameters in AAV production using VPC was the agitation speed. For instance, low agitation could increase cell clumping, reduce cell growth, and decrease AAV production significantly. The high agitation speed of 210 rpm enabled high AAV production and minimal cell aggregation.

Another important parameter is the high consumption of glucose and GlutMAX due to the fast cell growth and high AAV productivity. In the biomanufacturing process developed in this study, the same basal medium was used from Day -1 when seeding the production bioreactor until the end of AAV harvest without medium exchange or culture dilution. The batch culture of AAV production showed significant cell viability dropping on Day 2 (data not shown). The booster and enhancer added during transfection could extend the culture longevity and maintain high viability. However, it was found that more than 8.2 g/L of glucose was consumed from Day -1 to Day 3. Lack of assay to titrate GlutaMAX in culture broth, we assumed the 1:1 consumption rate of glucose and GlutaMAX, and fed 3.5 mM of GlutaMAX together with ~ 3.5 g/L of glucose between Day 1 and Day 2 to avoid nutrient depletion in this study. To further optimize AAV production, a full extracellular and intracellular metabolite analysis is needed to monitor glucose and GlutaMAX consumption and correlate cellular metabolism to cell growth and AAV production.

In the presented AAV bioproduction, we stopped culture at 72 h post triple-plasmid transfection, but AAV-DJ did not reach maximal value at the harvest viability. Therefore, we suspected that AAV titer could be further improved by optimizing the endpoint of production process via evaluating different harvest viabilities.

### Further optimization of AAV purification

The challenge in the purification of engineered AAV capsids is the lack of a high-specificity of binding resin with high capture rate. The generic IEX column separation developed in this study can be applied to multiple AAV serotypes, but the purity could be lower than the affinity column purification. To further improve the purity of AAV, affinity-based primary capture and purification followed with secondary or polishing strategies could be developed in future to benefit the recovery and purity of multiple AAV serotypes.

In addition, the primary purification using IEX aPrime 4A liquid chromatography column captured 85–95% AAV in one round of sample loading. To achieve higher capture rate, the loading capacity, flow rate of loading buffer, and packing strategy of purification resin should be further optimized. Another strategy is to run serial purification using both aPrime 4A and HPQ columns to improve the binding rate of AAV.

Ultrafiltration could further purify the AAV post IEX purification by removing the impurities with molecular weight of < 100 kDa, and also combine desalting, buffer exchange and sample concentration into one step. However, we observed that AAV-DJ had high retention rate in PES membrane but showed high recovery in regenerated cellulose column. Evaluation and selection of suitable ultrafiltration material might be needed for different serotypes. An alternative strategy is to use G25 desalting column or dialysis in combination with vacuum concentration to process the purified AAV, but the multi-step operation could reduce the recovery rate of AAV.

## Conclusions

AAVs have been widely used to deliver therapeutic genes for disease treatment or deliver the genes of interest for long-term transient expression in basic research due to the advantages of high infection and stable transient expression. This study reported a robust, scalable and suspensive biomanufacturing of multiple AAV serotypes, including stirred-tank bioreactor production and scale up to achieve high productivity, liquid chromatography purification and scale up to get high recovery rate, and post purification handling and evaluation of AAV quality. As compared to previously established AAV bioprocess, this advanced biomanufacturing can be easily adapted to GMP facility for large-scale production and purification. Moreover, this generic biomanufacturing can be used to produce and purify AAVs with different serotypes although fine adjustment or modification is needed. In addition to the high productivity and high recovery, the generated AAV demonstrated high function and quality. In conclusion, the biomanufacturing platform developed in this study could benefit the production, clarification and purification of AAV for basic research, pre-clinical study, translational research or clinical application.

## Materials and methods

### Triple plasmids for AAV construction

The AAV2, 5, DJ and DJ8 serotype-specific Rep-Cap plasmids, pHelper plasmid, and AAV-MCS Promoterless Expression Vector were purchased from Cell Biolabs (San Diego, CA, USA). The CMV promoter, luciferase reporter gene (*Luc*) and synthesized gene of interest (total of 3.9 kb) [25, 26] were cloned to construct pAAV expression plasmid following our previous publication [25]. These triple plasmids were used to transfect host cells for AAV production and evaluations in this study.

### Cells, media and cultures

All AAV producing host cells, culture media and nutrients were purchased from Gibco (Buffalo, NY, USA) and general supplies were purchased from Fisher Scientific (Waltham, MA, USA) unless otherwise specified. The Viral Production Cells 2.0 (VPC, Gibco, USA) were engineered and cloned from parental cell line HEK 293F, then well adapted to chemically defined Viral Production Medium. The seed train of VPC cells were cultivated in basal production medium supplemented with 4 mM of GlutaMAX in 125 or 250-mL shaker flasks at 125 rpm on an orbital shaker (Thermo Fisher Scientific, Waltham, MA, USA) with 19‑mm shaking diameter. HEK 293F cells were grown in FreeStyle 293 Expression Medium with 4 mM of GlutaMAX in shaker flasks at the same conditions of VPC cells. The small-scale (30 or 60 mL) VPC or HEK 293F host cells were maintained at 37 °C, 8% CO_2_ and 125 rpm in CellXpert™ incubator (Eppendorf, Enfield, CT, USA) for AAV production. The human glioblastoma cell line U251 (MilliporeSigma, Burlington, MA, USA) was cultivated in DMEM/F12 medium with 10% fetal bovine serum, 4 mM L-glutamine, 1% non-essential amino acids (NEAA), 1 mM sodium pyruvate, and 1% penicillin/streptomycin (100 IU/100 µg/mL) in T-75 flask at 37 °C and 5% CO_2_ [27]. The U251 cells were used for in vitro AAV transduction evaluation, gene expression analysis, and glioblastoma intracranial xenograft mouse model development for in vivo evaluations. The cell growth was monitored in terms of viable cell density (VCD) and viability using TC20 automated cell counter (Bio-Rad, Hercules, CA, USA).

### AAV production in shaker flask, spinner flask and bioreactor

*AAV production using HEK 293F* As described in our previous study [25], HEK 293F host cells were transfected with three plasmids with plasmid DNA:HEK 293F ratio of 1.6 μg:10^6^ cells, cationic liposomes as transfection reagent, and VCD of 0.4 × 10^6^ cells/mL. The transfection liposomes were synthesized using 1,2-dioleoyl-sn-glycero-3-phosphoethanolamine and 1,2-dioleoyl-3-trimethylammonium-propane [28]. The suspensive AAV production was carried out in 30 mL of culture using 125-mL shaker flask at 125 rpm, 37 °C and 5% CO_2_, or 100 mL of culture using 250-mL spinner flask at 210 rpm, 37 °C and 5% CO_2_. Raw AAV was harvested from the transfected HEK 293F cells at 40–60 h post transfection for further clarification and purification.

*AAV production using VPC* The viral production medium supplemented with 6 g/L of glucose and 4 mM of GlutaMAX was inoculated with VPC cells at a seeding VCD of 1.5 × 10^6^ cells/mL and incubated for 24 h to reach a VCD of about 3.0 × 10^6^ cells/mL before transfection. The key optimized transfection parameters include VPC density of 3.0 × 10^6^ cells/mL, pAAV expression plasmid: pAAV Rep-Cap: pHelper of 1:1:3, DNA: cell of 0.5 µg:1 million cells, 10% (v/v) viral-plex buffer, 0.6% AAV-MAX transfection reagent, 0.3% booster, and 1% enhancer (Gibco). Interestingly, the VPC cells showed poor growth in bioreactor at agitation speed of 130 rpm but reached high VCD and viability when the agitation was increased to 210 rpm. The AAV production process was evaluated at different scales: 1) 30 or 60 mL in 125 or 250-mL shaker flask at 37 °C, 125 rpm using shaker with 19-mm shaking diameter and 8% CO_2_, 2) 60–100 mL in 250-mL spinner flask at 37 °C, 210 rpm and 8% CO_2_, and 3) 1.2–2.0 L of working volume in 2.5-L stirred-tank bioreactor (Distek, North Brunswick Township, NJ, USA) with process controls of 37 °C, pH 7.0, 210 rpm and DO 40%. VPC cells were harvested at 72 h after transfection using centrifugation at 1,000 g for 10–20 min at 4 °C and cell pellets were stored at -80 °C for further purification and characterization.

### AAV clarification

The VPC cells were re-suspended in PBS buffer with 1/30 of the production volume, then raw AAV was released by adding 10% AAV-MAX lysis buffer (Gibco), 2 mM MgCl_2_ and 90 U/mL benzonase (Millipore Sigma). An alternative strategy was to directly lysis cell culture broth using the same formulation. The lysis mixture was incubated at 37 °C for 2–3 h on an orbital shaker or operated with three cycles of freeze in ethanol/dry ice for 30 min and thaw at 37 °C in water bath for 15 min. After a full cell lysis was confirmed with observation under microscope, the cell lysate was centrifuged at 13,000 g for 10 min or 4,500 g for 30 min at 4 °C. The supernatant containing AAV particles was collected and filtered by 0.45 μm and 0.22 μm regenerated cellulose membrane (serotype of DJ) or PES membrane (serotypes of 2, 5 and DJ8) to remove cell debris for clarification.

### AAV purification

To develop a universal primary purification method for multiple AAV serotypes (AAV2, 5, DJ and DJ/8), Bio-Rad NGC system equipped with four chromatography columns, including EconoFit Nuvia aPrime 4A ion exchange (IEX, anionic) column (Bio-Rad), Foresight Nuvia HPQ anionic column (Bio-Rad), HiTrap Q Sepharose XL anionic column (Cytiva, Marlborough, MA, USA), and HiTrap AVB Sepharose affinity column (Cytiva), for AAV purification. Among these columns, aPrime 4A anionic column achieved high recovery rate and purity for all four serotypes with equilibration buffer A (25 mM Tris–HCl, 20 mM NaCl, pH 9.0) and stepwise (0, 15, 25, 70, 85 and 100%) elution buffers of A and B (25 mM Tris–HCl, 1 M NaCl, pH 9.0) at flow rate of 1.0 mL/min. HPQ anionic column-based purification procedure generated medium level of recovery rate and purity of all four serotypes using equilibration buffer A (25 mM Tris–HCl, 20 mM NaCl, pH 9.0) and gradient (stepwise, 0, 50, 65 and 100%) elution buffers of A and B (25 mM Tris–HCl, 1 M NaCl, pH 9.0) with flow rate of 1.0 mL/min. The equilibration buffer of 20 mM Tris–HCl, 0.5 M NaCl, pH 8.0 and elution buffer of 0.1 M Glycine–HCl, 0.5 M NaCl, pH 2.5 with flow rate of 0.5 mL/min were used for HiTrap AVB Sepharose column. The HiTrap Q Sepharose anionic column was used for AAV purification with loading buffer of 25 mM Tris–HCl, pH 9.0 and elution buffer A of 25 mM Tris–HCl, pH 9.0 and buffer B of 2 M NaCl in 25 mM Tris–HCl, pH 9.0 with flow rate of 1.5 mL/min. The primarily isolated AAV was further purified using 100 kDa MWCO PES ultrafilter (AAV2, 5 or DJ8) or regenerated cellulose ultrafilter (AAV-DJ) to remove the small-size impurities. Then the purified samples were desalted using HiTrap G25 desalting column (Cytiva) with NGC liquid chromatography system (Bio-Rad) or 20 kDa MWCO slide-A-lyzer dialysis cassettes through buffer exchange. Finally, the AAV samples were concentrated using Savant SpeedVac (Fisher), or 10-kDa regenerated cellulose concentrator for AAV-DJ or PES concentrator for AAV 2, 5 and DJ8. The purified, desalted and concentrated AAV was sterilized using 0.22 µm filter, then stored in a formulation buffer composed of 1 × PBS, 5% Sorbitol, and 350 mmol/L NaCl at -80 °C for long-term storage.

### AAV titration

The AAV samples collected from raw cell lysate, post purification using ion exchange columns and ultrafilters, and post desalting and concentration were aliquoted and diluted with PBS for qPCR titration. First, the possible nucleotide contaminant was removed from the single-stranded AAV DNA by mixing 5 μL of AAV sample with 5 μL of DNase I Buffer (10X), 100 U of DNase I (336 U/μL), 1 U of Exonuclease I (20 U/μL), and UltraPure DNase/RNase-free distilled water (Gibco) in a 50-μL mixture. The digestion reaction was processed in a thermal cycler at 37 °C for 60 min, 85 °C for 20 min, and 4 °C until stop. The AAV samples digested with DNase were further processed to remove protein contaminant by adding 1 μL of Proteinase K and 49 μL of Proteinase K buffer (2X), followed with incubation in thermal cycle at 60 °C for 60 min, 95 °C for 10 min, and 4 °C until stop. The extracted ssDNA samples were 1:50 diluted with DNase/RNase-free distilled water. Second, the pAAV expression plasmid containing NLuc was linearized to prepare standard samples by serially diluting it to gene copy of 2 × 10^8^, 10^7^, 10^6^, 10^5^, 10^4^ and 10^3^ per μL in Eppendorf tubes with DNase/RNase-free distilled water. Third, the qPCR reaction was prepared by mixing PowerUp SYBR Green Master Mix (Applied Biosystems, Waltham, MA, USA), 250 nM of NLuc forward primer (5’-ATTGTCCTGAGCGGTGAAA-3’) and reverse primer (5’-CACAGGGTACACCACCTTAAA-3’), and UltraPure™ DNase/RNase-Free Distilled Water to reach volume of 15 μL each reaction. Fourth, 5 μL of AAV samples or standard samples were added into 96-well plate (Avantor, Radnor, PA, USA), followed with adding 15 μL of qPCR reaction mixture into each well. The 96-well plate was covered with an adhesive film and spin down with centrifugation at 1000 g for 1 min. The qPCR assay was performed in an Azure Cielo 96-well Real-Time PCR instrument (Azure Biosystems, Dublin, CA, USA) at 50 °C for 2 min, 95 °C for 2 min, and 40 cycles at 95 °C for 15 s and 60 °C for 1 min. Finally, the fluorescence was measured at 60 °C and data analysis was implemented by the Azure Cielo manager software (Azure Biosystems) to calculate the copy number of vector genome (vg).

### SDS-PAGE and Western blotting

NuPAGE 4–12% Bis–Tris protein gels (Life Technology, Carlsbad, CA, USA) were used to run non-reducing SDS-PAGE to characterize AAV. The gels were stained with Pierce Silver Stain Kit (Fisher) and imaged by Azure 300 biosystems (Azure Biosystems). To confirm the expression of capsid proteins of the produced AAV, the primary rabbit polyclonal anti-VP1/2/3 antibodies ordered from American Research Products Inc (Waltham, MA, USA) and HRP-conjugated secondary anti-rabbit antibody (Abcam, Cambridge, UK) were used for the immunodetection of VP1 (87 kDa), VP2 (73 kDa) and VP3 (62 kDa). The blotted PVDF membrane was treated with Luminata Forte Western HRP substrate (Millipore, Boston, MA, USA) and imaged by Azure 300 biosystems following our previously established protocol [29–31].

### Live-cell confocal imaging

The three-color confocal microscope imaging was performed to confirm the transduction capability and biological function of produced AAV following our previously reported procedure [32–34]. Specifically, the purified AAV was stained with Sulfo-cyanine 5.5 (Cy5.5) fluorescent dye (Lumiprobe, Cockeysville, MD, USA) following the manufacturing protocol. The unlabeled free dye was removed using 100 kDa MWCO PES concentrator using PBS with dilution factor of 1:10 for 5 times. The chambered glass coverslip was seeded with glioblastoma U251 cells at cell density of 1 × 10^5^ cells/mL, stained with BacMam GFP (Fisher) for cytoplasm detection, and incubated at CO_2_ incubator for overnight. The U251-GFP cells were stained with DAPI to image nucleus and transduced with AAV-Cy5.5 at multiplicity of infection (MOI) of 5,000 for 24 h. After washing out the free AAV and dye, the live-cell confocal images of stained U251 were captured using Echo Revolve fluorescent microscope (Echo, Cerritos, CA, USA) with fluorescent light cubes of FITC, DAPI and Cy5 to detect GFP, DAPI, Cy5.5, respectively. The transduction of AAV was evaluated by the overlap of green GFP (U251 cells), blue DAPI (nucleus), and red Cy5.5 (AAV).

### Transmission electron microscope (TEM) imaging

TEM images of AAV particles were collected following our previous procedure with modification [35]. Our AAV samples (3 µL) were negatively stained with 2% uranyl acetate on the glow discharged carbon grid purchased from Electronic Microscope Sciences (Hatfield, PA, USA) and incubated at room temperature for 1–2 min, followed with blotting off the stain. Tecnai F20 XT transmission electron microscope (Field Electron and Ion Company, Hillsboro, OR, USA) equipped with three CCD camera was used to collect images of AAV. Briefly, the AAV samples were loaded in DT rod first. Then both microscope alignment and fine alignment of gun and aperture were performed or confirmed before recording images. Finally, Gatan digital micrograph was captured with the WA-Orius camera.

### Xenograft model

The 6-week-old NSG (NOD.Cg-Prkdc < scid > Il2rg < tm1Wjl > /SzJ) mice (Jackson Laboratory, Bar Harbor, ME, USA) were used to generate glioblastoma orthotopic xenografted model following our previously established protocol [27, 36] with modification and approved IACUC protocol. Briefly, about 0.5 × 10^6^ U251 cells suspended in 3 μL of sterile saline buffer were intracranially implanted into the frontal region of cerebral cortex (2 mm lateral, 1 mm anterior and 1.5 mm ventricle of bregma) at rate of 0.4 μL per minute using Stoelting Just for Mouse Stereotaxic Instrument (Stoelting, Wood Dale, IL, USA). The burr hole in skull was closed with sterile bone wax deposited by rubbing wax back and forth from the wooden end of a sterile cotton-tipped applicator. The NSG mice received 5 mg/kg of carprofen via subcutaneous (s.v.) injection immediately before surgery and every 12–24 h for 48 h post-surgery. Bupivacaine stock of 2.5–5 mg/mL was topically administered with dosage of 1 mg/kg at the incision site during surgery.

### IVIS and bioluminescent imaging

The functional expression of AAV-delivered NLuc gene was tested in vitro using U251 cell line and in vivo using U251 cell line-derived intracranial xenografted NSG mouse model, respectively. For in vitro evaluation, 5 × 10^4^ cells/mL of U251 cells were used to seed 96-well plates and transduced with AAV at different MOIs, i.e. 1,000 to 7,500. Then 25 µM of substate ViviRen (Fisher) was added to the cells expressing AAV-delviered genes three days after transduction. The bioluminescence generated by the expressed NLuc protein was monitored with In Vivo Imaging System (IVIS) Lumina Series III (PerkinElmer, Waltham, MA, USA) [33]. The 6-well plate was seeded with 5 × 10^5^ cells/mL of U251 cells, transduced with AAV at different MOIs of 0, 1 × 10^4^ and 1 × 10^6^, and induced with 25 µM of substate ViviRen. The dynamic bioluminescence signal was detected with SpectraMax iD3 plate reader (Molecular Devices, San Jose, CA, USA). To detect in vivo expression of AAV-delivered NLuc gene, the U251 xenografted NSG mice received 1 × 10^10^ vg of AAV and 37 μg of ViviRen substrate through intracranial injection at the same coordinate of cells xenograft. The NLuc expression (i.e. bioluminescence) was detected in live animals using IVIS Lumina.

### Statistical analysis

All experimental data were presented as average ± standard deviation (STDEV) with replication number of 3. Two-tailed Student’s *t* tests with statistical significance of *P* value < 0.05 were used to determine the probability of significance between conditions.

## Data Availability

All data generated or analyzed during this study are included in this article.

## Abbreviations

AAV: Adeno-associated virus
VPC: Gibco viral production cells 2.0
VCD: Viable cell density
Agt: Agitation
DO: Dissolved oxygen
IEX: Ion exchange
